# Early identification of delayed-onset ADA deficiency: The case for expanded first-tier newborn screening

**DOI:** 10.70962/jhi.20250102

**Published:** 2025-11-10

**Authors:** Kristine Jeganathan, Adam Byrne, Gabrielle White, Eyal Grunebaum, Elie Haddad, Michael S. Hershfield, Teresa K. Tarrant, Matthew P.A. Henderson, Anne Pham-Huy

**Affiliations:** 1 Department of Pediatrics, Children’s Hospital of Eastern Ontario, University of Ottawa, Ottawa, Canada; 2 Division of Allergy, Immunology and Infectious Diseases, Children’s Hospital of Eastern Ontario, University of Ottawa, Ottawa, Canada; 3 Division of Immunology and Allergy, The Hospital for Sick Children, Toronto, Canada; 4 Department of Pediatrics, Department of Microbiology, Immunology and Infectious Diseases, CHU Sainte-Justine Azrieli Research Center, University of Montreal, CHU Sainte-Justine, Montreal, Canada; 5 Department of Medicine, Division of Rheumatology and Immunology, Duke University School of Medicine, Durham, NC, USA; 6 Department of Biochemistry, Duke University School of Medicine, Durham, NC, USA; 7 Durham Veterans Administration Hospital, Durham, NC, USA; 8 Newborn Screening Ontario, Children’s Hospital of Eastern Ontario, Ottawa, Canada

## Abstract

Delayed-onset ADA deficiency was identified in two patients who were not detected by standard newborn screening for SCID. These findings support expanding first-tier newborn screening to include purine profiling alongside TREC assays, ensuring early diagnosis and enabling curative treatment before irreversible end-organ damage develops.

## Teaser

Standard newborn screening using T cell receptor excision circles (TRECs) failed to detect delayed-onset ADA deficiency in two cases. Retrospective mass spectrometry (MS/MS) analysis showed diagnostic deoxyadenosine elevation at birth. We advocate integrating MS/MS purine profiling into first-tier screening to enable early diagnosis and timely curative treatment.

## Introduction

Adenosine deaminase (ADA) is a critical enzyme in purine metabolism, essential for lymphocyte production. Autosomal-recessive ADA deficiency causes severe combined immunodeficiency (SCID), marked by depletion of T, B, and natural killer (NK) cells, due to the accumulation of adenosine and deoxyadenosine (dAXP) metabolites. ADA SCID occurs in ∼1 in 200,000 live births, accounts for 10–15% of all SCID cases, and can be detected by newborn screening (NBS) for SCID using T cell receptor excision circles (TRECs) quantification from dried blood spots (DBS). Low or absent TRECs indicate impaired thymic output and abnormal T cell development. Early detection leads to improved outcomes with curative hematopoietic stem cell transplantation (HSCT) or gene therapy. In addition, enzyme replacement therapy (ERT) with a PEGylated recombinant ADA enzyme can also be used as bridging therapy to stabilize patients and improve immune function prior to curative treatment (1).

15% to 20% of ADA deficiency patients exhibit a delayed- or late-onset combined immune deficiency (CID). In these individuals, T cell production may be sufficient at birth, leading to TREC levels above the screening cutoff. Lymphocyte production in these patients declines with age. The diagnosis is made after the newborn period and typically only after recurrent infections, autoimmunity, or other manifestations of ADA deficiency occur, by which time end-organ damage may be irreversible (1).

Tandem mass spectrometry (MS/MS) is widely used for metabolic screening due to its high throughput, cost-effectiveness, and ability to measure multiple biomarkers simultaneously. More than 15 years ago, MS/MS methods were developed in Italy to detect adenosine and deoxyadenosine metabolites in DBS, showing superior sensitivity over TREC assays for delayed-onset phenotypes. (2). In Ontario, Canada, SCID NBS began in 2013 (3) using a two-tier strategy: first-tier TREC screening followed by MS/MS purine profiling if TRECs are below cutoff. This approach misses ADA-CID cases with normal TREC at birth. Here, we present two such cases and discuss implications for optimizing screening strategies.

## Case 1

Patient 1 is a 6-year-old boy referred to the immunology clinic for evaluation after 2 years of persistent, nonallergic rhinitis and chronic sinusitis, resistant to standard therapies. He was born at term to non-consanguineous Caucasian parents of European descent, with normal growth and development. There were no recurrent or severe infections prior to age 4. NBS at birth showed a TREC value of 89 copies/3 µl, exceeding the Ontario cutoff (75 copies/3 µl), and no further testing was performed.

At age 6, he developed a single dermatomal, vesicular rash consistent with uncomplicated shingles. He had previously received the live-attenuated measles-mumps-rubella and varicella vaccines without adverse events. Immunologic tests at presentation showed protective vaccine responses and normal serum immunoglobulins. However, he had lymphopenia with nearly absent B cells, reduced T cells, and very low NK cells. (Table 1). HIV serology was negative. Genetic testing identified heterozygous pathogenic ADA variants (NM_000022.4) inherited in trans: c.476G>A (p.Arg156His) and c.218+1G>A (predicted to affect splicing). The p.Arg156His variant has specifically been associated with a delayed-/late-onset phenotype (4). ADA1 enzyme activity was undetectable, and total dAXP nucleotides were markedly elevated (0.293 μmol/ml RBC: normal <0.002), with dAXP representing 13.8% of total red cell adenosine nucleotides (normal < 0.2), consistent with ADA deficiency with a delayed-onset phenotype. Retrospective analysis of his birth DBS (tested >5 years after collection) revealed increased levels of deoxyadenosine (5.3 µmol/liter, normal: 0.1–0.4), consistent with ADA deficiency.

**Table 1.** Findings at time of diagnosis

​	​	Reference values	Patient 1	Patient 2
Initial immune evaluation	CD3	1,600–6,700 × 10^6^/L	350	239
	CD4	1,000–4,600 × 10^6^/L	129	179
	CD8	400–2,100 × 10^6^/L	256	55
	CD19	600–2,700 × 10^6^	4	14.4
	CD (15 + 56)	200–1,200 × 10^6^/L	9	47
	RTE	900–5,800 × 10^6^/L	20	30
	PHA (CD3 percentage)	≥60.8%	29.6% of control	Not done
	IgG	1.3–6.8 g/L	9.2	9.5
	IgA	≤0.2 g/L	0.5	0.57
	IgM	0.2–0.9 g/L	0.53	2.5
	IgE	≤36 µg/L	93.4	93
	Anti-tetanus toxoid IgG	<0.10 IU/ml indicates no detectable antibodies present	1.89 IU/ml	0.49 IU/ml
	Anti-diphtheria toxoid IgG	<0.10 IU/ml indicates no detectable antibodies present	0.19 IU/ml	0.17 IU/ml
Age of onset of symptoms		​	4 years	6 mo
Past infections at presentation	​	​	Cellulitis, bullous impetigo, sinusitis, and 1 episode of shingles	Recurrent sinopulmonary infections
				3 hospital admissions (between ages 8–11 mo):
				Influenza A+ with superimposed bacterial pneumonia and bilateral AOM
				Coronavirus OC43+ with pneumonia requiring i.v. antibiotics and oxygen supplementation
				Rhinovirus+ with superimposed pneumonia requiring i.v. antibiotics and high-flow nasal cannula oxygen therapy
Hearing impairment		​	None	None
Other complications		​	None	Suspected pulmonary alveolar proteinosis (chronic lung changes)
Neurodevelopmental complications		​	None	None
Age at diagnosis		​	6 years	11 mo
dAXP (% dAXP) at diagnosis (Duke University)		<0.002 µmol/L (<0.2%)	0.293 µmol/L (13.8%)	0.358 µmol/L (18.1%)
AXP at diagnosis (Duke University)		1.465 ± 0.38 µmol/L	1.829 µmol/L	1.625 µmol/L
Deoxyadenosine at diagnosis (NSO)		0.1–0.4 µmol/L	15.1 µmol/L	Not done

NSO, newborn screening Ontario; AXP, total adenosine nucleotides; % dAXP = [dAXP]/[AXP + dAXP] × 100; RTE, recent thymic emigrants; PHA, phytohemagglutinin (T cell mitogen used in lymphocyte proliferation assays).

He was started on *Pneumocystis jirovecii* pneumonia (PJP) prophylaxis, immunoglobulin replacement therapy (IgRT), and ERT with elapegademase (Revcovi). At the age of 7 years, he underwent a matched sibling HSCT, leading to successful immune reconstitution and discontinuation of ERT and IgRT. He had no reported hearing impairment, neurodevelopmental complications, or other organ involvement at the time of follow-up.

## Case 2

Patient 2 is a 1-year-old boy, born at term to consanguineous Yemeni parents. He had a history of recurrent, severe respiratory infections starting at age 6 mo and requiring three hospital admissions by age 11 mo. Evaluation revealed failure to thrive, chronic lung changes, lymphopenia, neutropenia, hypergammaglobulinemia, and profound lymphopenia of T, B, and NK cells (Table 1). HIV serology was negative. Similarly, NBS for SCID, collected at birth, was negative with 582 copies/3 μl—well above the cutoff; therefore, second-tier purine profiling was not performed. Retrospective MS/MS analysis of his birth DBS revealed elevated deoxyadenosine levels (6.88 µmol/liter, reference interval 0.1–0.4), consistent with ADA deficiency.

Genetic testing identified homozygous ADA pathogenic variants (NM_000022.4): c.385G>A (*p.Val129Met*), also previously associated with a delayed-onset phenotype (5). ADA activity was undetectable, and dAXP was elevated (0.358 μmol/ml RBC).

Following diagnosis, PJP prophylaxis, IgRT, and ERT were initiated. At the age of 23 mo, he underwent a haplo-identical sibling HSCT, resulting in full chimerism. ERT and IgRT were discontinued. Prior to transplant, there were chronic lung changes suggestive of possible pulmonary alveolar proteinosis that improved following transplant. However, some chronic changes persisted, likely as a reflection of his recurrent pneumonias prior to diagnosis. He was neurodevelopmentally normal with preserved hearing at follow-up.

## Discussion

These two cases highlight the limitations of relying exclusively on TREC values at birth for the diagnosis of ADA deficiency. Both patients had TREC levels above the set cutoff at birth, leading to missed early diagnoses and delayed intervention. Diagnosis was made following symptomatic disease. Retrospective MS/MS analysis of DBS samples obtained at birth revealed elevated deoxyadenosine, diagnostic for ADA deficiency, suggesting that first-tier MS/MS testing would have enabled earlier diagnosis and avoidance of infectious complications.

MS/MS is already integrated in NBS for many inborn errors of metabolism but has not yet been widely adopted for first-tier testing for ADA deficiency and other purine-related disorders. Some individuals with ADA deficiency may remain undiagnosed well into childhood, often presenting with chronic and recurrent infections, pulmonary disease, and autoimmune manifestations, and may already have irreversible organ damage. Early diagnosis permits timely initiation of ERT and curative therapies, with overall reduction in morbidity. While expanded first-tier MS/MS screening would improve case detection, concerns about overdiagnosis, especially in milder or asymptomatic cases, must be addressed. Improved genotype–phenotype characterization, such as the variant-activity correlations reported by Santisteban et al., could help distinguish patients likely to benefit from early intervention (6). While the reagent cost for purine profiling is relatively low, the total implementation cost is difficult to estimate due to added expenses related to sample retrieval, confirmatory testing, and follow-up coordination. Notably, Azzari et al. estimated the reagent cost of adding purine metabolite analysis to existing MS/MS platforms to be ∼USD 0.013 per sample, with preliminary studies showing high diagnostic accuracy (2). Although ADA SCID treatment, including ERT, can be costly, sometimes exceeding hundreds of thousands of dollars per year, early detection and prompt initiation of therapy are critical to prevent toxic metabolite accumulation and irreversible organ damage. While economic factors may influence how screening programs are implemented, expanded first-tier NBS, incorporating purine profiling alongside TREC assays, would improve early identification of infants with ADA deficiency and facilitate timely initiation of definitive therapy while potentially reducing the overall duration of ERT.

Wilson and Jungner’s criteria for effective screening are largely met by MS/MS screening for delayed-onset ADA deficiency. ADA deficiency is a serious, treatable condition; suitable tests are available (MS/MS), effective treatments exist, and early diagnosis can prevent serious complications. However, uncertainties remain regarding the natural history of delayed-onset ADA deficiency and determining which infants require therapy versus observation. Further research is needed to address these concerns.

## Conclusion

These cases highlight that reliance on TREC values alone fails to detect ADA deficiency when T cell output at birth is sufficient—as in delayed-onset forms. While earlier studies have evaluated MS/MS for ADA deficiency detection in pilot settings, our study offers real-world evidence from Canada, where MS/MS is not currently a part of first-tier NBS. Retrospective MS/MS analysis showed that deoxyadenosine levels were already markedly elevated at birth, supporting the integration of purine profiling with TREC assays in first-tier screening to improve early detection of ADA deficiency, particularly in cases with delayed onset. We recommend heightened clinical vigilance for immune defects missed by standard SCID NBS and support further research into the feasibility, cost, and impact of MS/MS-based purine profiling to enhance early detection and reduce the burden of ADA deficiency.

## Statement

According to the CHEO Research Institute “Guidance on Case Report” policy (CHEO RI, 2025), case reports involving three or fewer patients that arise from routine clinical practice and lack research intent or systematic investigation do not require research ethics board review. Consent for publication was obtained from the guardians for all cases included in this report.
